# German first-year medical students’ expectations of their professional life – concerns and hopes: A project report

**DOI:** 10.3205/zma001654

**Published:** 2023-11-15

**Authors:** Karen Schmidt-Bäse, Johanna Huber, Martin R. Fischer, Marjo Wijnen-Meijer

**Affiliations:** 1 Technical University of Munich, TUM School of Medicine, TUM Medical Education Center, Munich, Germany; 2 LMU Hospital, LMU Munich, Institute of Medical Education, Munich, Germany; 3 Technical University of Dresden, Medical Faculty Carl Gustav Carus, Dresden, Germany

**Keywords:** medical students, work-life balance, physician's role, survey

## Abstract

**Aim:**

Expectations among medical students towards their future professional life are affected by intrinsic and extrinsic factors which may change during years of medical school. The aim of this study is to gain further insight into students’ expectations of their professional life at the beginning of medical school. Findings regarding contextual influences can be used to improve curricula and student guidance.

**Methods:**

The project report based on an online survey among three cohorts of first year medical students at the LMU. The questionnaire consisted of six open-ended questions which addressed the student’s motivation, expectations, anticipations and concerns of their professional life. Questions were also asked about presumed personal development and influence on private life. An inductive coding was used in this qualitative content analysis.

**Results:**

Written responses from 591 participants were coded, categorized and summarized into four main themes: personal life, work, science, personal issue. Despite coming from different cohorts, the occurrence of the main themes showed the same trend in all student groups. Students are worried most about the work-life-balance, and they expect it to be a difficult issue. But many of our first-year students are optimistic that they will be able to establish a good work-life-balance or that the working conditions will have changed to a manageable workload at the time they will enter their first jobs. The majority of our students expect to become more self-confident with enhanced empathy and team-working ability and more patient and stress-resistant in daily challenges.

**Conclusion:**

The medical students emphasize the gender-neutral desire for work-life balance. So, they expect improved working conditions for the future – an ongoing challenge for the health care system.

## Zusammenfassung

**Ziel:**

Erwartungen von Medizinstudierenden an ihr zukünftiges Berufsleben werden durch intrinsisch und extrinsisch Faktoren beeinflusst, die sich im Laufe des Medizinstudiums ändern können. Ziel dieser Studie ist es, weitere Erkenntnisse über die Erwartungen der Studierenden an ihr Berufsleben zu Beginn des Medizinstudiums zu gewinnen. Erkenntnisse über kontextuelle Einflüsse können genutzt werden, um Curricula und Orientierungshilfen für Studierende zu verbessern.

**Methodik:**

Der Projektbericht basiert auf einer Online-Befragung von drei Kohorten von Medizinstudierenden, die sich jeweils im ersten Studienjahr an der LMU befanden. Der Fragebogen bestand aus sechs offenen Fragen, die sich mit der Motivation, den Erwartungen, Vorfreude und Sorgen der Studierenden in Bezug auf ihr Berufsleben befassten. Gefragt wurde auch nach der vermuteten Persönlichkeitsentwicklung und dem Einfluss auf das Privatleben. Bei dieser qualitativen Inhaltsanalyse wurde eine induktive Kodierung verwendet.

**Ergebnisse:**

Die schriftlichen Antworten von 591 Teilnehmern wurden kodiert, kategorisiert und in vier Hauptthemen zusammengefasst: Privatleben, Arbeit, Wissenschaft, persönliche Aspekte. Das Auftreten der Hauptthemen zeigte in allen Studierendengruppen den gleichen Trend, obwohl die Studierenden aus unterschiedlichen Kohorten stammten. Die Studierenden machen sich am meisten Sorgen um die Work-Life-Balance und erwarten, dass dieses ein schwieriges Thema sein wird. Aber viele unserer Studienanfängerinnen und -anfänger sind optimistisch, dass sie in der Lage sein werden, eine gute Work-Life-Balance zu etablieren oder dass sich die Arbeitsbedingungen zum Zeitpunkt ihres Eintretens in das Berufsleben auf ein überschaubares Arbeitspensum geändert haben werden. Die Mehrheit unserer Studierenden erwartet selbstbewusster zu werden mit verbesserter Empathie und Teamfähigkeit, sowie geduldiger und stressresistenter in täglichen Herausforderungen zu werden.

**Schlussfolgerung:**

Die Medizinstudierenden betonen – geschlechtsneutral – den Wunsch nach einer Work-Life-Balance. Für die Zukunft erwarten sie daher bessere Arbeitsbedingungen – eine anhaltende Herausforderung für das Gesundheitssystem.

## Introduction

All medical students have expectations of their future profession as a doctor, the doctor’s role and reputation and perceived hindrances in medical work. These expectations may be affected by various factors, such as labor market conditions, parental career expectations [1], [2] demographic changes, developments in technology, personal attitudes and their experiences during medical school. A set of important intrinsic and extrinsic factors have been subject of research, and it has been showed that the perspective on some aspects change during the years going from medical school to residency [3], [4], [5], [6]. For medical schools and the community of medical professionals, it is important to understand students’ expectations, so we can better respond and consider them. A previous study about medical students’ expectations found that (mis)perceptions had an impact on students’ performance and confidence [7].

The future shortage of medical doctors, especially in family medicine, is a major concern not only in low- and middle-income countries but also in western high-income countries [8], [9]. Reasons such as generational changes and gender aspects are being discussed [4], [10].

The young generation show different attitudes towards work compared to older colleagues: *“a well-balanced integration of professional and private life is an essential goal for the new generation of doctors”* [11]. Key intrinsic contributors to work-life-balance are enjoyment, meeting work requirements and time management [6]. The balance is shaped by extrinsic factors such as peer groups, family and professional culture.

Diderichsen et al. [10] layed out that there is not only the home/family domain but also a leisure domain (physical activity, time with friends and hobbies) which must be balanced. Other aspects may be as well important: *“living with a partner seems to influence both male and female students in their preferences”* …. (…for speciality choice – added by the authors) [3], [6]. This change of preferences might be seen as a personal development since (personal) life events have been shown by several studies to be crucial for personal development [12].

Since the public perception of the so-called “feminization” of medicine (based on an increase of female medical students) raises, gender aspects in motivation of choosing medical study or preferred medical specialties gain more and more attention. Women and men have different career goals and motives: men value profession-related motives (e.g. income, status) higher, whereas women value life-style motives (like flexible working hours, control over their lifestyle) higher than men [3]. The eventual choice of specialty is affected by various factors, gender being one [6]. To recognize that women show remarkably more positive attitude toward family medicine than men might help to fight the feared future shortage of family practitioners [9], [13], [14].

*“Accurate knowledge of medical students’ preferences for future specialty, their flexibility regarding location and workload, and their views on the compatibility of work and family life is essential for planning of provision.”* [5]. For medical teachers it is important to understand students’ expectations, so they can better support the transition into the reality of (clinical) practice e.g. by development of mentoring-programs. In addition, our results may be valuable for developing new seminars in the area of ethics in medicine or professional identity formation.

The aim of this project report is to give further insight into students’ expectations of their professional life at the beginning of medical school. Understanding the expectations of beginning medical students provides the opportunity to address that in education.

## Methods

Our study is part of a multi-centric study at five medical schools in Europe. The aim of the international project is to gain further insight into the contextual factors which shape students’ expectations. Incoming students are asked to complete an electronic open-ended questionnaire during the first weeks of medical school with participation being voluntary. The students will be asked to complete the same task at the end of medical school to consider the degree of which expectations change over time. The study will be repeated for five successive cohorts. This enables the researchers to identify the effect of medical studies on students’ perceptions of their experiences and on possible changes in attitudes related to health care. In addition, each university will keep a logbook tracking the influence of internal and external contexts on students’ expectations. The study runs in five locations (besides Munich, these are: Stockholm, Leeds, Utrecht, Nicosia). The study started in 2017 and will run for 10-12 years. It originated as a joint research project of participants in an international exchange programme.

Our study here uses data collected via a student survey in Munich (using survey tool “Unipark”-ESF Survey Software). In Munich, all medical students start together at the Ludwig-Maximilians-University (LMU) for the first two years. For the following clinical training they may either switch to the TU Munich or stay at the LMU. Three cohorts of first year medical students were asked to complete an electronic open- ended questionnaire during the first weeks of medical school with participation being voluntary. Anonymity is guaranteed: each participant receives a generated code which will be stable for each person. Assignment of answers to persons is not possible. The survey consists of two sections. The first section covers demographic information (gender, age, questions for pseudonymisation). These demographic data were analyzed with Microsoft-Excel to ensure a comparable composition of the cohorts. The second section asks the following six questions:

1. Why do you want to be a doctor?
2. What are your expectations of your future profession as a doctor?
3. What do you look forward to in your future profession as a doctor?
4. What do you not look forward to?
5. In your perception: how will working as a medical professional influence you as a person and your private life?
6. What questions are coming up thinking about your professional future?

Our survey is an enhancement of the survey used in the multi-centric study stated above: Only the first four questions are part of the questionnaire used by the other universities. Question 5 and 6 were only asked in our study in Munich. Question 5 was aimed to investigate possible implications on the students’ personality development. Question 6 may provide information on future fears and hopes associated with either the personal situation or the upcoming developments in the health-care sector. For all six questions we use solely open-ended questions in the hope that this may enable us to detect new themes which are important for the students’ ideas about their future.

This paper reports findings from the first three rounds of data collection from 2019 to 2021.

The project was conducted in German. Responses were translated to English by two researchers working independently. In this way the data are accessible for the international researchers. We used an inductive coding in our qualitative content analysis. Responses were coded line by line in an open coding process according to the wording and their semantic content, followed by axial coding to identify main categories. To reveal which code was most prevalent, the number of responses of each code was counted (data not shown). The data were analyzed as a group. We grouped the identified codes into 11 categories, which were summarized into four main themes. For each question (Q1-Q6) the three or four most important themes are shown in table 1 (Tab. 1).

## Results

### Demographic data

We invited 918 students in year 2019, 921 in year 2020 and 847 in year 2021 to participate in the survey. The questionnaire was answered by 144, 283 and 181 students, respectively. That gave a response rate of 15,6% for 2019, 30,7% for 2020 and 21,4% for 2021.

At least one open question was answered by 139 (2019)/245(2020)/ 178 (2021) students. Agewas not stated by 11 (2019), 16 (2020) and 10 (2021) students, respectively. In 2019 and 2020 more than two thirds of the participating students were women which is a 6% higher ratio than published for all beginners in medical school [15] in Germany in these two years (see table 2 (Tab. 2)). In 2022, the female portion of our participating students is more than 10% higher than in the former years (no German-wide data published so far).

The median age was roughly the same for the first two cohorts (21 years) but only 20,5 years in 2021. In the first two cohorts about 1/6 of the students start medical school at an age of 25 years or older, while in 2021 there were fewer “older beginners”. The majority of the “older beginners” are women. This is in accordance with the overall gender ratio mentioned above for the 2020 and 2021 cohorts, while in 2019 the portion of male “older beginners” is about 15% higher than the overall male ratio (see table 2 (Tab. 2)). The answers to the questions varied in length from a few words to several sentences. 

### Personal life

Comparing the occurrence of the main categories indicated that “work-life-balance” occurs within answers to four out of the six open-ended questions: the students are worried about the work-life-balance and they expect it to be a difficult issue. For example:

*“I believe that the private life will be restricted in a certain way and especially working in a hospital will not match my work-life-balance but I think this profession will be very fulfilling.” (Student – female 2019, Q5).*

*“A very demanding and exhausting but also fulfilling profession which is nevertheless compatible with family life.” (Student – male 2021, Q2).*

They expect it will be a rewarding job in a respected and financially secure (Q1-3) work field.

*“A very diversified and exciting job in a very satisfying environment, job security, appropriate compensation and many career options.” (Student – male 2021, Q2)*.

The assumptions about the personal development are divers: few students presume “no personal change”, the majority expects to become more self-confident with enhanced empathy and team-working ability and also becoming more patient and stress-resistant in daily challenges.

*“In addition I assume, that the requirements will help me to get resistant against fatigue and stress.” (Student – male 2019, Q5).*

*“I will become more open and understanding and will gain more self-confidence.” (Student – female 2021, Q5).*

### Work

They are seriously concerned about excessive bureaucracy and not having enough time for patients e.g., because of economic pressure (Q4).

*“I’m not up to the burden of bureaucracy and I don’t want to be restricted by external constrains how much time I spend with my patients.” (Student – female 2019, Q4)*

*“Matters of health shouldn´t be judged on the base of economic matters.” (Student – male 2021, Q4)*

Although being only in their first year the majority of students in year 2019 and about 30% in year 2020 and 2021 already think about the preferred specialty and workplace characteristics.

*“Will I really become the specialist as planned or will I do something totally different?” (Student – female 2019, Q6).*

Answers to Q4 indicated that there are severe concerns about “hierarchy” in the clinical setting and about “discrimination of women”.

*“I have no desire for the disadvantage facing women, also with regards to the desire to have children. I hope the sexism will disappear.” (Student – female 2021, Q4).*

The hierarchy aspect was remarkably more present in the group of older students: the number of mentions rises from 5% (young students’ group) to more than 15% (age >24 years; see table 3 (Tab. 3)).

Some female students fear discrimination based on the gender.

*“Will there be job models, which suits parents? Will women be promoted for higher positions, or will they still be dominated by men?” (Student – female 2021, Q6).*

*“I hope that women will treated equally in hospitals, as that was not the case in the courses I attended.” (Student – female 2021, Q2).*

Others mentioned future (technical) aspects driven by the spreading of AI in the medical field e.g. assistance systems. The students voiced hopes about improved working conditions in the future as a consequence of the pandemic. In contrast mentioning of pandemic specific vocabulary like “system-relevant” are very rare for all questions.

*“…crisis as the corona pandemic: how are these crises influence the job of a medical doctor?” (Student – female 2020, Q6).*

### Personal issue and Science

The theme “help people” was the most prevalent one for Q1, Q2 and Q3. The students are motivated to become doctors because they want to help people. At least one third of the students are very interested in science but only about 1/10 look forward to doing research (Q1+ Q2).

*“I see two reasons for becoming a medical doctor: 1. Great interest in natural science with a special focus on research. 2. I would like to work in an environment where I can help people actively and directly. Medicine is the only subject which combines both in an optimal way.” (Student – female 2019, Q1).*

The students are worried about making wrong decisions and asked themselves if they are good enough for this challenging job. 

*“Will I be good and intelligent enough? Can I stand the pressure?” (Student – female 2019, Q6).*

*“What will happen when I do mistakes? Will I deal with the responsibility correctly and will I be able to decide on my own?” (Student – male 2021, Q6)*

Despite coming from different cohorts, the occurrence of the main themes showed the same trend in all student groups. The students are able to differentiate positive and negative aspects of working as a medical doctor.

## Discussion

In this project report we describe an online-survey among three cohorts of first-year medical students on their expectations of their future professional life and possible shaping factors. 

### Personal life

Our students are worried most about the work-life-balance, and they expect it to be a difficult issue. In line with other research [5], [10], we found that this desire for work-life-balance is gender-neutral.

Today’s students need to be balanced the home/family domain with the leisure domain. The priorities may change over the course of medical school: Life events such as living with a partner, getting married or having children affected the perspectives on work-life-balance [3], [6] as well as the (speciality) choices for the future, see below.

In this context it is important to note that the presumed possibility to balance work and private life is one of the facilitators attracting medical students to become a family practitioner [9], [11], [16]. Consequently, future doctors should be aware of the importance of their personal well-being to deliver effective care. Especially the handling of one’s own emotions seems to be a challenge for young physicians [17]. But in a British study less than half of the 137 medical students reported receiving support learning self-care [6]. Therefore, the authors recommended that teaching staff in medical schools should emphasize self-care and support

the development of time management skills. In contrast to this, many of our first-year students are optimistic that they will be able to establish a good work-life-balance or that the working conditions will have changed to a manageable workload at the time they will enter their first jobs.

### Personal issue and Science

The majority of students start medical school because they want to help people. A number of studies have found that students tend to lose empathic and idealistic motivations during their medical studies [18], [19]. They become more cynical and less idealistic towards patients and the profession. Some studies identifying the beginning of the decline in the third year of US curricula, (the time when the students are exposed to clinical life) while the study of Morley [20] suggests that the decline begins already in the first two years: several items that represent idealistic motivations decrease and items like money, lifestyle, career increase as medical careers move forward. The decline may also contribute to the observation, that there is a dramatic loss of interest in family medicine during the second year of undergraduate studies. If this correlation holds, we will need interventions to preserve the idealism and to train enough students in family medicine [5]. The Canadian researchers attributed this to the curriculum on one hand and to the “cultural influences” (Hidden curriculum) [19], [21] based on derogating comments about family medicine by faculty teaching staff on the other hand. This might result in challenges for recruitment in those specialties like family medicine [22].

A systematic review suggests that educational interventions can be effective in maintaining and enhancing empathy in (undergraduate) medical students [18]. Writing interventions for example, where students write essays from the patient’s point of view, can improve medical (written) skills on certain affective dimensions [23], [24]. Further research is necessary to clarify best practices. Also, the gender-specific effects of different medical humanities programs still require further studies [25]. Looking at the teaching methodology of these possible interventions it might be advisable for medical educators to follow Ainoda [26]: although self-directed learning (SDL) has been widely used in medical education, mainly in the scientific-technical dimension – *“from a patient-centred viewpoint, socio-emotional goals should be stressed more in self-directed learning …for developing patient-physician communication skills or fostering ethical or altruistic attitudes”*.

Early studies attributed vanishing interest in service to underserved populations or to the community to the above-mentioned decrease of idealism during medical school [27], [28]. In contrast Morley [20] and others recommended explicit these activities as possible alternative interventions. Interestingly, volunteering students during the first wave of the pandemic in 2020 uncover both altruistic and introjected motivations – *“Sense of duty and the desire to help were…the most important reasons for volunteering”* [29]. All volunteering students were already in the clinical phase.

Although at least one third of our students is very interested in science, only about 1/10 look forward to doing actual research. In contrast, in 2018 a survey of pharmacy students from 13 German universities showed that 26,6% of those students intended to work in science and university teaching [30]. There is a distinct need for translating scientific findings in medicine into application. To promote this a number a clinician scientist programs have been established in the last 10 years (status 2021: 72 programs at 39 locations in Germany [31]).

These programs allow a “protected research time” during residency years. Extensive foundation by the DFG underlines the importance of these programs [32]. To prepare the research-interested physicians for a long-term academic career more programs at advanced levels emerged in recent years (e.g. BIH Charite’ Clinician Scientist Program; DZIF- Academy). Since 2019 clinician scientist professorships are supported by the Else Kröner Stiftung to raise the attractiveness of this vocational path [https://www.ekfs.de/en/suche?search_api_fulltext=clinician%20scientist].

### Work

The hierarchy aspect was much more present in the group of older students. Although this was not explicitly questioned in the survey, we conclude from a high number of answers to the open questions that these students were already trained in the medical (or science) field prior to joining a medical school. Therefore, we speculate that there is a correlation between already existing experience in the clinical work field and the mentioning of “hierarchy”. Lind et al. [33] identified entrenched hierarchies and power differentials as key factors that contribute to student’s perceived mistreatment during medical school. The position of a student in the clinical setting is often weak because he/she is not in the position to decide on or perform the necessary activity in patient care. This may result in demotivation *(“not feeling like an important part of the clinical team”* [34]) and can be defined as suboptimal learning environment [33].

Suboptimal learning environments like *“significant lack of confidential and non-punitive feedback from faculty members after minor mistakes”* may finally result in decreased patient safety because the important speak-up culture has not been learned [33], [35].

Interestingly, the experience of feeling useful and of belonging to the community of health care workers was positively reported from medical students who volunteered in the hospital during the first wave of the COVID-19 pandemic in 2020 [36], [37].

Some women express concerns of possible gender-based discrimination in their future professional life. During medical school there is already a high amount of student’s perceptions of mistreatment which varied significantly by gender and race-ethnicity [38]. In addition, there is a high variability in the students’ perceptions of mistreatment within specialty departments: the overall treatment in family medicine was significantly better than in all other departments. In paediatrics women perceive better treatment than men while in surgery women reported unfavourable treatments. This may have an influence not only on learning during medical school but also on the specialty choice in later years. The reported occurrence of negative treatments increased with the number of years of study [7], [34] and women were more serious and longer disturbed by the treatment. Comparing different faculties, these mistreatments were interestingly mostly present in the faculties of medicine and education [34] – in both professional areas most of the students are female. It might be important for the development of a professional doctor-patient treatment what atmosphere has been experienced by the future doctors themselves.

### Personal development 

Other findings [7], [39] illustrate that medical school constitutes a strong socializing experience. *“The teaching atmosphere … is important not only for learning but also for building positive attitudes towards one’s professionals’ identity …”* [34]. The majority of our students expect to become more self-confident with enhanced empathy and team-working ability and more patient and stress-resistant in daily challenges. These hopes confirm the findings of Gasiorowski [7] who noted positive changes consisting of enhanced maturity and self-confidence in final year medical students. … *“which are likely to have an impact on future doctors’ professional practice.”* [7]. This seems also true for other health professions like occupational therapy: a number of external (e.g. clinical experience, relationships with peers, staff and patients), and internal aspects (such as certain personal characteristics) have been determined which influence the important foundation of professional confidence during student years [40].

The start of working life might promote personality maturation since increased conscientiousness, extraversion and agreeableness have been found in young adults [41].

Asselmann et al. [41] speculated that subjective perceptions of the new role lead to increased personal investment and finally to a higher commitment to the new social role. This “…might trigger adaptational processes toward a more “mature” behaviour …” [41] and leads to the above-mentioned increase in conscientiousness and agreeableness. This fits to the findings of Picton [6]: year 5 medical students anticipated a less work-heavy balance than year 3 students do. Picton guess that this may be due to an enhanced role understanding of the more mature students. But it could also be due to emotional distancing from work as an inner act of protecting oneself [42].

At this point it is interesting to note the experiences of those medical students who volunteered to help at the hospital during the first wave of the COVID-19 pandemic: students had benefited in their personal and professional development. They reported a largely positive impact on their professional identification and confirmed their career choice [36], [37]. It is possible that this is a result of different complementary factors:

Wundrack et al. [12] investigated the impact of personal versus collective life events on personal development: *“…personal life events usually imply the adaption of a new social role, while collective life events can also change the requirements of existing social roles by changing the social practices and meaning around them (=culture).”* [12]. In sum, this would explain why the students no longer experienced their position as being “weak” in the clinical setting as mentioned earlier: cultured change in the clinical teams (driven by the collective life event COVID-19 pandemic) together with the demand of taking a new social role as a real team member (based on the personal life event “volunteering”) allows the reported personal maturation.

This assumption is underlined by a cross-cultural examination of young adults from 62 nations that showed significant cultural differences regarding the moment the personality maturation occurs [42], [43]. *“Consistent with social-investment theory, results showed that cultures with an earlier onset of adult-role responsibilities were marked by earlier personality maturation.”* [43].

## Limitations

Our study has some limitations: the mean response rate of 22,6% is low and varied enormous between the three years (SD 6,2). The clearly highest response rate in 2020 (30,7%) might be due to the fact, that these beginners started medical school at that time where teaching has shifted to 100%- online teaching. Our study used solely open-ended questions. It is possible that this contributes to the low response rate, considering the time it takes to answer compared to fixed answers to choose among. Since the comparison of demographic characteristics showed an over-representation of female students, there might be a gender-related bias in the answers. The answers to question 5 indicate that our wording in question 5 is probably not precise enough: here many answers deal only with the “private life” and do not pay close attention to the personal development. Our study was conducted in one German university and our data may not generalize to experiences of students at other German universities. However, first data from other European countries reported similar main themes for the first four questions [44].

## Conclusions

Our report provides interesting data regarding the expectations and fears of Munich medical students in their first year. First findings of contextual influences were identified and will be further examined in the following cohorts and at different points in time to improve curricula and student guidance. Some critical aspects might be seen in another light using the multifarious positive experiences from the voluntary work during the first wave of the pandemic. This may allow us the advice to medical educators to integrate more “service learning-like-interventions” into the curricula of medical schools. 

The future physicians emphasize the gender-neutral desire for work-life balance. This will be a challenge for health care workforce planning. We are eager to see how the mentioned future aspects “AI”, “working conditions” and “improvement of working conditions because of the pandemic” develop in the next years respectively the next cohorts.

## Ethical approval

Approval for the study was obtained from the Ethics Committee of the Faculty of Medicine of the Technical University of Munich (approval no. 439/19s). The survey was anonymous and voluntary, and all participants gave consent in accordance with the Declaration of Helsinki.

All students received information on the nature, purpose and procedure of the survey and their right to withhold or revoke their consent at any time.

## Acknowledgements

We thank the students Dorine Hamann und Elena Pepeldjiyska (LMU Munich) for data collection and primary coding. We gratefully acknowledge the support of Michael Meyer, student dean of the faculty of medicine, LMU Munich.

## Competing interests

The authors declare that they have no competing interests. 

## Figures and Tables

**Table 1:**
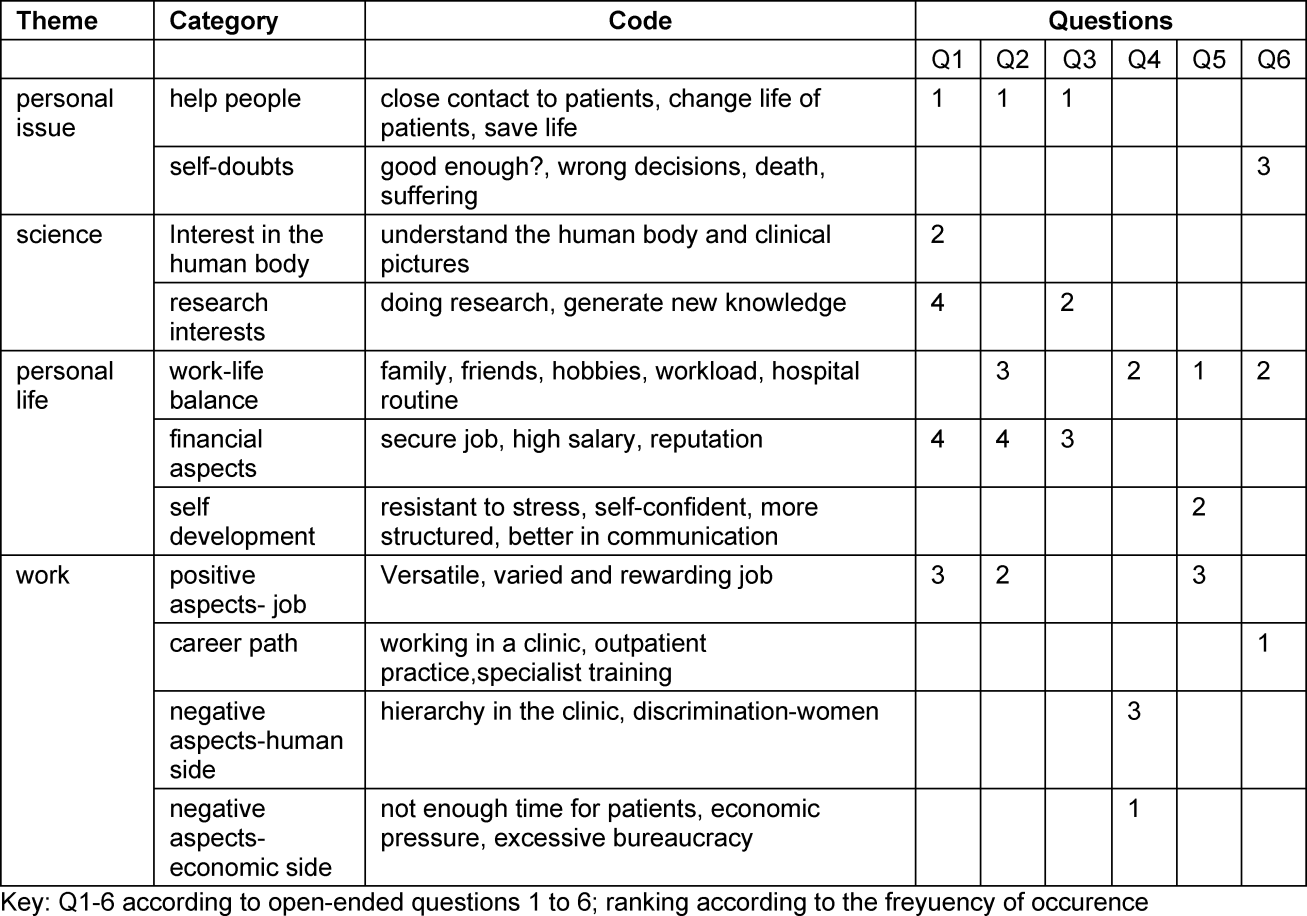
Main themes, categories and distribution of answers according to quotations

**Table 2:**
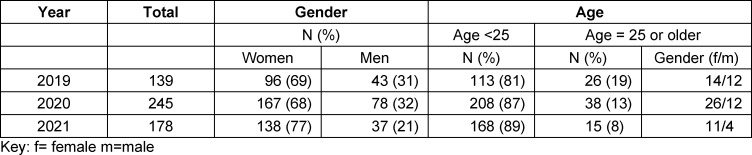
Demographic data of students with at least one open question answered

**Table 3:**
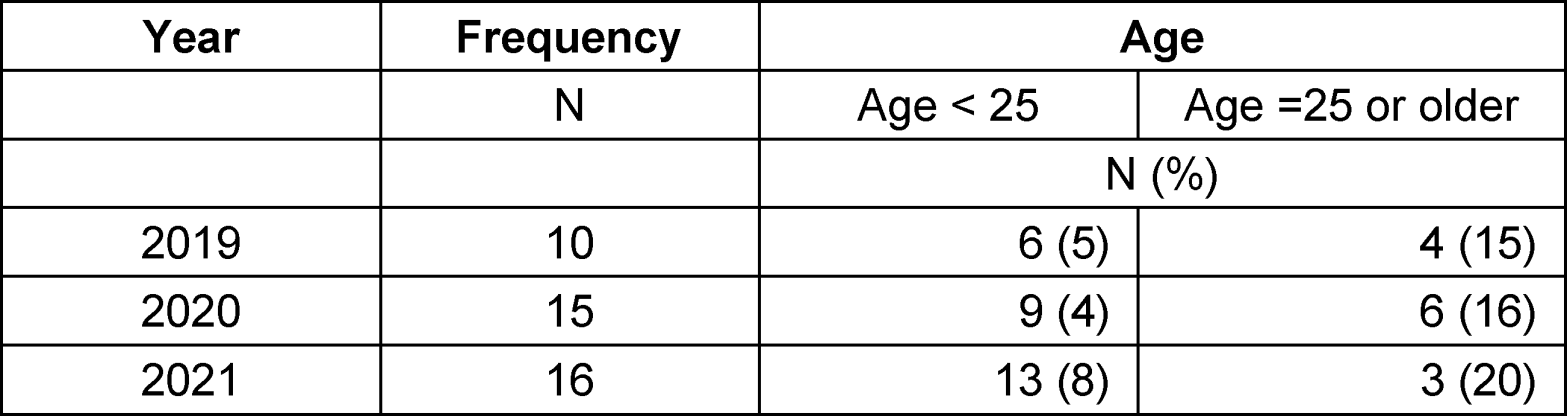
Responses mentioning hierarchy aspect
